# Vocal cord dysfunction diagnosis may be improved by a screening questionnaire

**DOI:** 10.1186/2045-7022-5-S2-P12

**Published:** 2015-03-23

**Authors:** Marcelo Vivolo Aun, Lucia Helena Eduardo Pinto, Jorge Kalil, Rosana Câmara Agondi, Pedro Giavina-Bianchi

**Affiliations:** 1 Clinical Immunology and Allergy Division, University of Sao Paulo School of Medicine, Sao Paulo, Brazil; 2 University of Sao Paulo School of Medicine, Clinical Immunology and Allergy Division, São Paulo, Brazil

## Background

Many patients with vocal cord dysfunction, with or without asthma, receive inappropriate treatment because they are misdiagnosed as having difficult-to-control asthma alone. We developed a clinical screening questionnaire designed to aid the diagnosis of vocal cord dysfunction.

## Method

A prospective observational study involving 80 patients aged ≥ 18 years, diagnosed with severe asthma. After anamnesis, physical examination, and application of a questionnaire with 6 questions to identify vocal cord dysfunction.<TABLE01> Then patients underwent spirometry and laryngoscopy. On the basis of the laryngoscopic findings, we created three patient groups: vocal cord dysfunction (vocal cord adduction during inspiration, n=14); unconfirmed vocal cord dysfunction (inconclusive findings, n=29); and control (normal findings, n=37). We attempted to determine whether any of those groups were associated with the responses to individual questions or sets of questions on the questionnaire.

## Results

The proportion of affirmative answers to the question “Does pulmonary auscultation reveal wheezing predominantly in the cervical region, or stridor?” was significantly higher for the vocal cord dysfunction group than for the other two groups (*P*=0.006). The control group was significantly different from the other two groups in terms of the variable “4 or more affirmative answers” (*P*=0.022).

## Conclusion

A finding of wheezing or stridor on auscultation of the cervical region is suggestive of vocal cord dysfunction, especially in elderly patients, and such dysfunction can be confirmed through laryngoscopy. Our screening questionnaire proved effective in discriminating between patients with vocal cord dysfunction and those without.

